# Different Selectivity in Fungal Communities Between Manure and Mineral Fertilizers: A Study in an Alkaline Soil After 30 Years Fertilization

**DOI:** 10.3389/fmicb.2018.02613

**Published:** 2018-10-31

**Authors:** Ying Wang, Hongfei Ji, Yaxian Hu, Rui Wang, Junpeng Rui, Shengli Guo

**Affiliations:** ^1^State Key Laboratory of Soil Erosion and Dryland Farming on the Loess Plateau, Northwest A&F University, Yangling, China; ^2^Environmental Microbiology Key Laboratory of Sichuan Province, Chengdu Institute of Biology, Chinese Academy of Sciences, Chengdu, China

**Keywords:** fungal community composition, diversity, saprotrophs, Illumina HiSeq sequencing, long-term fertilization

## Abstract

Fertilizer application has contributed substantially to increasing crop yield. Despite the important role of soil fungi in agricultural production, we still have limited understanding of the complex responses of fungal taxonomic and functional groups to organic and mineral fertilization in long term. Here we report the responses of the fungal communities in an alkaline soil to 30-year application of mineral fertilizer (NP), organic manure (M) and combined fertilizer (NPM) by the Illumina HiSeq sequencing and quantitative real-time PCR to target fungal internal transcribed spacer (ITS) genes. The results show: (1) compared to the unfertilized soil, fertilizer application increased fungal diversity and ITS gene copy numbers, and shifted fungal community structure. Such changes were more pronounced in the M and NPM soils than in the NP soil (except for fungal diversity), which can be largely attributed to the manure induced greater increases in soil total organic C, total N and available P. (2) Compared to the unfertilized soil, the NP and NPM soils reduced the proportion of saprotrophs by 40%, the predominant taxa of which may potentially affect cellulose decomposition. (3) Indicator species analysis suggested that the indicator operational taxonomic units (OTUs) in the M soil occupied 25.6% of its total community, but that only accounted for 0.9% in the NP soil. Our findings suggest that fertilization-induced changes of total fungal community were more responsive to organic manure than mineral fertilizer. The reduced proportion of cellulose decomposition-related saprotrophs in mineral fertilizer treatments may potentially contribute to increasing their soil C stocks.

## Introduction

Fertilization has contributed substantially to increasing crop yield (Zhang et al., 2012). Long-term fertilizer inputs, especially nitrogen (N) inputs, can result in reducing soil pH (Guo et al., 2010) and increasing soil organic carbon (C) and total N content (Guo et al., 2011; Jian et al., 2016), which potentially influence soil fungal community composition. While soil fungi play important roles as decomposers (Fontaine et al., 2011; Ma et al., 2013), plant symbionts (Clemmensen et al., 2006) and pathogens (Ohm et al., 2012), the effects of fertilizer application on fungal communities have not yet well understood.

Previous research on N fertilization and fungal community structure mostly focused on total fungal community (Weber et al., 2013; Yuan et al., 2013; Wang et al., 2017) or specific subsets of fungi, e.g., mycorrhizal fungi (Gryndler et al., 2006; Sheng et al., 2013; Ekblad et al., 2016) and saprotrophic fungal taxa (Allison et al., 2007). Only few studies comprehensively describe the fungal community in a given ecosystem (Sterkenburg et al., 2015; Morrison et al., 2016), and most of them mainly focused on N fertilization in acidic or neutral soils. For example, in addition to descriptions of entire community and phyla over discussions of species, some reports have shown that mineral N application has a positive impact on soil saprotrophic fungi in soils of pH 4.5–6.8 (Sterkenburg et al., 2015; Morrison et al., 2016) and promotes fungal genera with known pathogenic traits (Hartmann et al., 2015; Paungfoo-Lonhienne et al., 2015; Zhou et al., 2016). While soil pH in acidic and neutral soils is very susceptible to N application and in turn affects fungal community composition (Zhang et al., 2016) and decreases fungal diversity (Zhou et al., 2016), soil pH in alkaline soil is less affected by N fertilization due to its higher pH buffering capacity (Guo et al., 2010). Thus, in alkaline soil, fungal community might be more linked to changes of soil nutrients rather than soil pH (Lauber et al., 2008). However, the potential effects of fertilizer on total fungal community and major fungal functional groups in alkaline soil have been less explored (Rousk et al., 2010). In alkaline soil, N application has been found no influence on fungal community structure (Mueller et al., 2015; Chen et al., 2016) but increasing fungal class Sordariomycetes (Mueller et al., 2015). In fact, alkaline soil is widely distributed in northern China, especially on the Loess Plateau with great relevance for regional and national food security (Kuhn et al., 2016). Furthermore, organic amendments have been reported to increase soil nutrient status and organic C content (Jian et al., 2016), but the response of fungal communities and functional groups to manure application has not been thoroughly studied (Francioli et al., 2016). This therefore calls for a systematic investigation on the possible impacts of mineral fertilizer and organic manure treatments on soil fungal community structure and functional population dynamics in alkaline soils.

In this study, we collected alkaline soil samples from a 30-year fertilization experiment in northwest China and determined the abundance and community composition of fungi under mineral and organic fertilization. The Illumina HiSeq sequencing and quantitative real-time PCR of fungal internal transcribed spacer (ITS) genes were used to quantitatively and qualitatively assess changes in fungal communities. We hypothesized that long-term fertilization in alkaline soil not only shifts fungal community structure, but also changes the proportions of fungal functional groups, which may help us to advance our current understanding of fertilization introduced increase in soil fertility.

## Materials and methods

### Experimental site and soil sampling

The experiments were established in 1984 in the Changwu Agro-ecological Experimental Station on the Loess Plateau 107°40′E, 35°12′N, altitude 1,220 m, Shaanxi province, China. Detailed experimental design and management have been described in previous reports (Huang et al., 2003; Guo et al., 2011). In brief, this site has a semi-arid climate with an annual rainfall of 584 mm (1957–2001) and an annual average temperature of 9.1°C, representing a typical rain-fed agricultural area on the Loess Plateau and in the warm temperate zone of China. The soil is loam developed from loess deposits. It contains 6.5 g·kg^−1^ total organic C, 0.8 g·kg^−1^ total N and pH of 8.5 in 1984.

Four treatments were compared to investigate the effects of long-term mineral and organic fertilizer inputs on the soil fungal community. These treatments included control without fertilizer (NoF), organic manure (M), mineral fertilizer (nitrogen and phosphorus, NP) and mineral fertilizer plus organic manure (NPM) with a winter wheat (*Triticum aestivum* L.)-summer fallow rotation system. Fertilizer N and P were applied as urea (120 kg N ha^−1^ per year) and triple superphosphate (40 kg P_2_O_5_ ha^−1^ per year). Cow manure was applied at a rate equivalent to 87 kg N ha^−1^ per year. Each plot was 10.3 × 6.5 m in size, separated by 0.5-m buffer strips. Three replicated plots were sampled for each fertilizer treatment in May 2014 at a depth of 0–20 cm. Five cores were taken from each plot and homogenized to reduce within-plot variability. All samples were passed through a 2.0-mm sieve, stored at −80°C for DNA extraction and at 4°C for other analyses (the analyses were performed within 3 weeks of sample collection). Aboveground biomass was removed manually at harvest.

### Soil chemical and biological parameters

Soil pH was determined with a soil to water ratio of 1:5. Soil nitrate (${\text{NO}}_{3}^{-}$-N) and ammonium (together as soil available inorganic N, AN) were extracted from the soil by horizontal shaking with 2 M KCl (1:10) for 1 h and determined with a continuous flow analytical system (FLOWSYS, Italy). Soil available inorganic P (AP) was extracted with 0.5 M NaHCO_3_ and determined using the molybdenum blue method. Total organic carbon (TOC) was measured by the dichromate oxidation method and total nitrogen (TN) by the Kjeldahl method. Soil microbial biomass C and N (MBC and MBN) were measured following the chloroform fumigation-extraction method (Joergensen and Brookes, 1990) with 4 g soil and 16 mL 0.5 M K_2_SO_4_ extractant. No conversion factor of MBC and MBN was used because none had been determined for the soil used in this study.

Soil basal respiration was measured according to the method modified from Enwall et al. (2007). In brief, fresh soil (10 g) from each replicate was placed in a 125 mL glass bottle, sealed with a rubber plug. The soil was incubated at 25°C for 72 h. Headspace CO_2_ concentrations were measured via gas chromatography (HP7890A, Agilent Technologies, CA, USA). The respiratory quotient *Q*co_2_ was calculated by the ratio of soil respiration per day to microbial biomass C (Anderson and Domsch, 1986).

### Soil DNA extraction and quantitative real-time PCR (qPCR)

Soil DNA was extracted from 0.5 g soil using the FastDNA® Spin Kit for Soil (MP Biomedicals, Cleveland, OH, USA) and the FastPrep-24 instrument according to the manufacturer's instructions. The purified DNA was diluted with 100 μL sterilized water and checked for quality and quantity using a NanoDrop Spectrophotometer.

The abundances of bacterial 16S rRNA and fungal ITS genes were quantified using real-time PCR according to the method modified from previous studies (Fierer et al., 2005; Op De Beeck et al., 2014). PCR primers for bacterial 16S rRNA genes are 338F (5′-ACTCCTACGGGAGGCAGCAG-3′) and 518R (5′-ATTACCGCGGCTGCTGG-3′), and those for ITS genes are ITS3 (5′-GCATCGATGAAGAACGCAGC-3′) and ITS4 (5′-TCCTCCGCTTATTGATATGC-3′), which were targeted the ITS2 region. It should be caution when comparing 16S rRNA gene copies between treatments, since the 16S rRNA gene copies per cell vary between 1 and 15 (Hallin et al., 2009). Purified PCR products from a common DNA mixture (equal amounts of DNA from all samples collected in this study) were used to prepare sample-derived quantification standards described by Chen et al. (2007). The copy numbers of 16S rRNA and fungal ITS genes in each standard were calculated by DNA concentration (ng/μL) divided by the average gene molecular weight. The 25-μL qPCR reaction mixture contained 12.5 μL of SYBR® Premix Ex Taq^TM^ (Takara), 0.4 μL of each primer (10 mM) and 1 μL of 10-fold diluted soil DNA for bacterial and fungal quantification. QPCR amplification was performed in a thermal cycle equipped with a CFX96 Real-Time system (Bio-Rad). Duplicate technical replicates were performed for each sample. The reaction program was as follows: 95°C for 1 min, 40 cycles of 95°C for 15 s, 55°C for 30 s, and 72°C for 30 s. A dissociation step was added at the end of the real-time PCR to assess amplification quality. The specificity was further evaluated by running 20 randomly selected PCR products (for each gene) on a 1.5% (w/v) agarose gel.

### Illumina HiSeq sequencing of ITS gene amplicons and data analysis

DNA was amplified using the primers ITS3 and ITS4. Primers were tagged with unique barcodes (7 bp) for each sample. Both forward and reverse primers were tagged with adapters. PCR reactions were carried out in a 30-μL mixture with 15 μL of Phusion® High-Fidelity PCR Master Mix (New England Biolabs), 0.2 μM of each primer and approximately 10 ng template DNA. Thermal cycling was as follows: 98°C for 1 min; 30 cycles of 98°C for 10 s, 55°C for 30 s, and 72°C for 1 min; 72°C for 5 min. Sequencing was performed using the Illumina Hiseq2500 platform (250 bp paired-end reads were generated) by the Research and Testing Laboratory (Novogene, China). Sequence analysis was performed by the UPARSE software package using the UPARSE-OTU and UPARSE-OTUref algorithms (Edgar, 2013). Sequences with ≥97% similarity were clustered into operational taxonomic units (OTUs). The aligned ITS gene sequences were used for a chimera check using the Uchime algorithm (Edgar et al., 2011). Taxonomy was assigned basing on the Blast algorithm, which was calculated by QIIME software (Version 1.7.0) in combination with Unite database (https://unite.ut.ee/). Each sample was rarefied to the same number of reads (35,154 sequences) for both alpha-diversity (chao1 estimator of richness, observed species, Evenness, Shannon's diversity index and the Good's coverage) and beta-diversity (PCoA) analyses. The original sequence data are archived at the European Nucleotide Archive (ENA) with accession number PRJEB21749 (http://www.ebi.ac.uk/ena/data/view/PRJEB21749).

Generic identifications enabled us to classify OTUs into functional groups, for example as saprotrophs, ectomycorrhizal fungi, or pathogens (sensu Tedersoo et al., 2014; see Supplementary Table 3, Supplementary Information for functional classifications). All Glomeromycota were considered to be arbuscular mycorrhizal (AM) fungi.

### Statistical analysis

The differences in soil parameters and proportions of fungal taxa in the soil fungal community among treatments were tested by one-way analysis of variance (ANOVA) using GenStat. *Post-hoc* analyses were performed using Tukey's multiple comparison test at *P* < 0.05 (GenStat® for Windows 12.0; VSN Int. Ltd, UK). Correlations of soil parameters with fungal abundance, diversity and proportions of dominant fungal groups were analyzed by Spearman's correlation procedure using SPSS 17.0 software. To determine the relative importance of the various significant environmental factors in contributing to variation in the fungal abundance, diversity and proportions of dominant fungal groups across samples, we used a multiple regression model with backward selection procedure. Starting with the significant terms (*P* < 0.05) from Spearman's correlation, we removed variables one at a time; the differences in *R*^2^ values between each step were used to calculate the relative importance of the independent variable removed from the model. The environmental data were normalized using zero-mean normalization in the multivariate regression analysis and also the following redundancy analysis. The mean values, standard errors, and *P* values for these statistical analyses were based on four treatments in triplicate.

With the untransformed fungal proportion at the OTU level as input data, weighted Fast UniFrac distances between the samples were calculated and PCoA was performed on the basis of the distance measured using the WGCNA package (Langfelder and Horvath, 2008), stat package and ggplot2 package (http://ggplot2.org/) in R software (Version 2.15.3). Principal component analysis (PCA) and redundancy analysis (RDA) were carried out with Canoco 4.5 (Ter Braak and Smilauer, 2002). Monte Carlo permutation (999 repetitions) was used to test the relationships between the soil parameters and fungal communities. Significant differences in fungal community structure between treatments were determined by multi-response permutation procedures (MRPP) (*PC-ORD* 5.0, MjM software, www.pcord.com). To identify OTUs that were most responsible for changes in fungal community structure between fertilizer treatments, indicator species analysis (ISA) was conducted using untransformed fungal OTU proportion data in PC-ORD (De Cáceres and Legendre, 2009; Berry et al., 2012).

## Results

### Soil chemical and biological parameters

All measured soil parameters except pH were significantly affected by 30-year fertilizer applications (Table 1). The soil nitrate concentration was highest in the NPM and lowest in the NP, while the soil microbial biomass C and N were highest in the M and lowest in the unfertilized soil. Compared to the unfertilized soil, both mineral fertilizer and organic manure applications improved soil total organic C and total N concentrations, with greater increases in the M and the NPM than in the NP (Table 1). Soil respiration rates were also increased by manure applications (M and NPM). Overall, soil parameters showed the most pronounced differences between the unfertilized soil and all fertilized soils (Supplementary Figure 1), but MRPP analysis significantly differentiated all fertilized and unfertilized soils (Table 2).

**Table 1:** Chemical and microbial parameters in an alkaline soil under long-term fertilizer application.

**Soil parameter^§^**	**NoF^‡^**	**M**	**NP**	**NPM**
pH	8.68 ± 0.07 a^†^	8.71 ± 0.06 a	8.57 ± 0.02 a	8.59 ± 0.03 a
${\text{NO}}_{3}^{-}$-N (mg kg^−1^)	3.95 ± 0.53 b	3.58 ± 0.58 b	2.72 ± 0.15 b	7.18 ± 0.56 a
Available P (mg kg^−1^)	7.46 ± 1.01 c	34.63 ± 2.21 a	22.74 ± 1.6 b	33.67 ± 1.17 a
Total organic C (g kg^−1^)	6.60 ± 0.18 c	10.01 ± 0.38 a	7.91 ± 0.3 b	10.74 ± 0.2 a
Total N (g kg^−1^)	0.81 ± 0.03 b	1.22 ± 0.05 a	0.95 ± 0.04 b	1.33 ± 0.04 a
MBC (mg kg^−1^)	50.44 ± 3.75 b	104.54 ± 0.3 a	51.56 ± 4.31 b	62.01 ± 5.4 b
MBN (mg kg^−1^)	12.17 ± 0.49 c	20.55 ± 0.17 a	17.34 ± 0.1 b	18.53 ± 0.55 b
RP (mg CO_2_-C kg^−1^)	0.20 ± 0.01 c	0.34 ± 0 a	0.29 ± 0.01 b	0.36 ± 0 a
Crop biomass (kg ha^−1^)	4288 ± 30 c	9731 ± 27 b	10557 ± 119a	10590 ± 30 a

† *Values followed by same letter within a parameter are not significantly different at P < 0.05 (n = 3). Values are means of three replicates ± standard error*.

§ *MBC, microbial biomass C; MBN, microbial biomass N; RP, respiration rate*.

‡ *NoF, no fertilizer; M, organic manure; NP, nitrogen plus phosphorus; NPM, NP plus M*.

**Table 2:** Effect of fertilizer regimes on soil parameters and community structure of soil total fungi and fungal functional groups revealed by multi-response permutation procedure (MRPP) analysis.

**Comparison**	**Soil**		**Total fungi**		**Sarpotrophs**		**Plant pathogen**	
	***T***	***P***	***T***	***P***	***T***	***P***	***T***	***P***
NoF vs. M	−2.833	0.023	−2.871	0.022	−2.702	0.023	−2.747	0.023
NoF vs. NP	−2.811	0.023	−2.799	0.023	−2.758	0.023	−2.946	0.022
NoF vs. NPM	−2.884	0.022	−2.764	0.023	−2.843	0.022	−2.837	0.022
M vs. NP	−2.871	0.022	−2.945	0.022	−2.949	0.022	−2.792	0.023
M vs. NPM	−2.540	0.024	−2.884	0.022	−2.978	0.022	−2.794	0.023
NP vs. NPM	−2.737	0.023	−2.911	0.022	−2.849	0.022	−2.424	0.027

*T, test statistic; more negative, more separation between communities (NoF, no fertilizer; M, organic manure; NP, nitrogen plus phosphorus; NPM, NP plus M)*.

### Fungal abundance, alpha- and beta-diversity

The fungal ITS gene abundance was increased by fertilizer application compared to the unfertilized soil, with greater increases in the M and NPM than in the NP according to the qPCR analysis of fungal ITS genes (Table 3). The ratio of bacterial 16S rRNA to fungal ITS genes ranged from 22.8 to 33.6 in all the treatments, and it decreased in the M soil compared to the unfertilized soil.

**Table 3:** Bacterial 16S rRNA and fungal ITS gene copy numbers and fungal diversity indices in each treatment.

**Treatment^‡^**	**Bacteria^†^**	**Fungi^†^**	**Bacteria/fungi^‡^**	**Coverage**	**Chao 1**	**Simpson**	**Shannon**	**Evenness**
NoF	269.7 ± 14.8*c*^§^	8.88 ± 0.4c	30.8 ± 0.2a	0.996 ± 0a	810 ± 44.9a	0.77 ± 0d	5.97 ± 0.2b	0.96 ± 1.5b
M	458.9 ± 8.8b	18.87 ± 1.5ab	22.9 ± 1.0b	0.995 ± 0a	879.6 ± 22a	0.81 ± 0c	6.24 ± 0.1ab	1.45 ± 0.3b
NP	433.1 ± 3.9b	14.7 ± 1.1b	31.9 ± 1.1a	0.996 ± 0a	784.5 ± 6a	0.87 ± 0b	6.25 ± 0.1ab	1.01 ± 0.3b
NPM	708.6 ± 46.8a	21.13 ± 0.3a	33.6 ± 2.7a	0.996 ± 0a	846.3 ± 5.4a	0.9 ± 0a	6.71 ± 0.0a	5.62 ± 0.2a

§ *Values followed by the same letter within a parameter are not significantly different at p < 0.05 (n = 3). Values are means of three replicates ± standard error*.

† *Bacterial 16S rRNA and fungal ITS gene copy numbers were measured using qPCR. Bacteria/fungi: the ratios of bacterial 16S rRNA to fungal ITS gene copy numbers*.

‡ *NoF, no fertilizer; M, manure; NP, nitrogen plus phosphorus; NPM, NP plus M*.

A total of 541,482 high quality and chimera-free reads was obtained by Illumina HiSeq sequencing of fungal ITS gene amplicons with 35,154 to 59,139 reads per sample. The Good's coverage values were higher than 0.99 with a 97% similarity cutoff for all soils (Table 3), indicating that the number of sequence reads was sufficient to determine the fungal diversity in these soils. There were no significant differences in fungal richness (Chao1) or coverage between the fertilized and the unfertilized soils. However, Simpson and Shannon diversity indexes were generally higher in the fertilized than the unfertilized soil (Table 3). The fungal evenness was higher in the NPM than the other soils.

**Figure 1:**
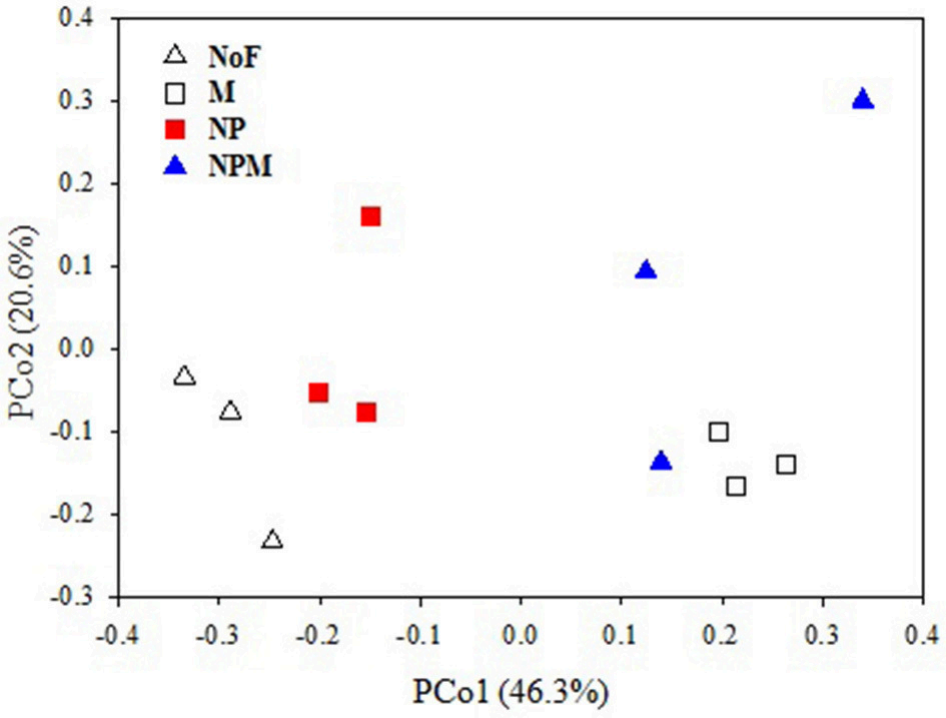
PCoA score plot of no fertilizer and fertilizer application soils in long-term trials based on weighted Fast UniFrac metric. Values on PCoA axes indicate the percentages of total variation explained by each axis. NoF, no fertilizer; M, organic manure; NP, nitrogen plus phosphorus; NPM, NP plus M.

The PCoA plot of the weighted Unifrac distances was used to assess the fertilizer application effects on soil fungal community. The first two principal coordinates represented 46.3% (PCo1) and 20.6% (PCo2) of the variation in fungal communities (Figure 1). PC1 generally separated fungal communities based on soil fertility indicators, i.e., total organic C and total N. The fungal communities from soils with relatively higher soil fertility (M and NPM) were located on the right, while those from soils with lower soil fertility (NP) were on the left. The unfertilized soil with the lowest values of soil fertility indicators was on the far left. The MRPP results further confirmed that fungal community structure significantly differed between the unfertilized and the fertilized soils, and also differed among fertilizer treatments (*P* < 0.05, Table 2).

### Taxonomic and functional composition of soil fungi

**Figure 2:**
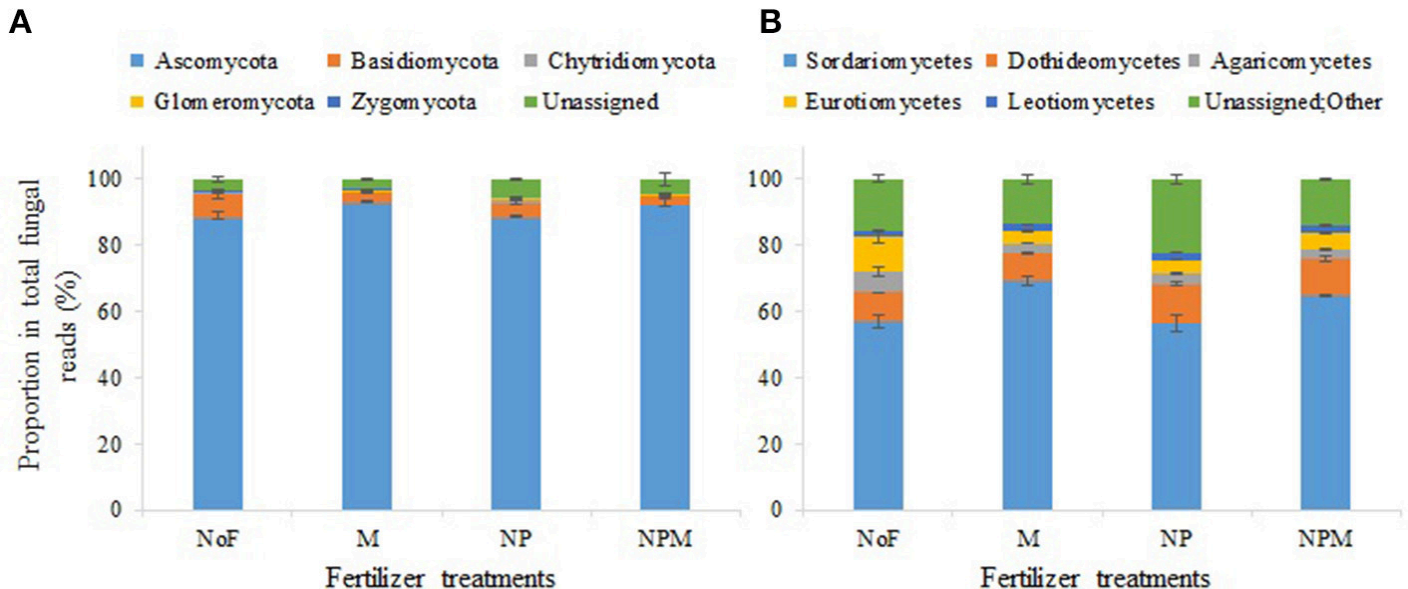
Soil fungal community composition under different fertilization regimes for 30 years in an alkaline soil. Proportions of fungal phyla **(A)** and classes **(B)** in the soil fungal community. Error bars are standard errors (*n* = 3). NoF, no fertilizer; M, organic manure; NP, nitrogen plus phosphorus; NPM, NP plus M.

In all 12 samples, Ascomycota predominated in the fungal community with a proportion of 84.1–93.7%, followed by Basidiomycota (2.7–9.9%), Chytridiomycota (0.03–0.7%), Zygomycota (0.2–0.5%) and Glomeromycota (0.1–0.4%) (Figure 2A). Both the mineral fertilized and the organic manure fertilized soils reduced the proportion of Basidiomycota compared with the unfertilized soil (Figure 2A, Supplementary Table 1). The most dominant classes (with read proportions higher than 1%) for all soils were Sordariomycetes, Dothideomycetes, Eurotiomycetes and Leotiomycetes (phylum Ascomycota), with proportions of 57.2–75.0%, 8.0–12.9%, 3.5–13.2%, and 1.4–2.5%, respectively, and Agaricomycetes (phylum Basidiomycota) (Figure 2B).

The most dominant identified genera were affiliated to phylum Ascomycota and showed different responses to fertilizer regimes (Supplementary Figure 2). The proportions of genera *Chaetomium, Penicillium*, and *Talaromyces* in total fungal reads were decreased by fertilizer applications by 73–93% (Supplementary Figure 2A,B). The genera *Staphylotrichum, Tetracladium*, and *Trichocladium* affiliated to phylum Ascomycota and *Cryptococcus* in phylum Basidiomycota generally had the highest proportion in the NP soil, and lowest proportion in the manure amended soils (M and NPM) (Supplementary Figures 2C–F). The proportion of genus *Kernia* was increased by manure amendment (M and NPM), with a greater increase in the M than in the NPM. The proportion of genera *Pseudogymnoascus* and *Fusarium* was increased by fertilizer application, with greater increases in the NP and NPM soils than in the M soil (Supplementary Figure 2G–I).

**Figure 3:**
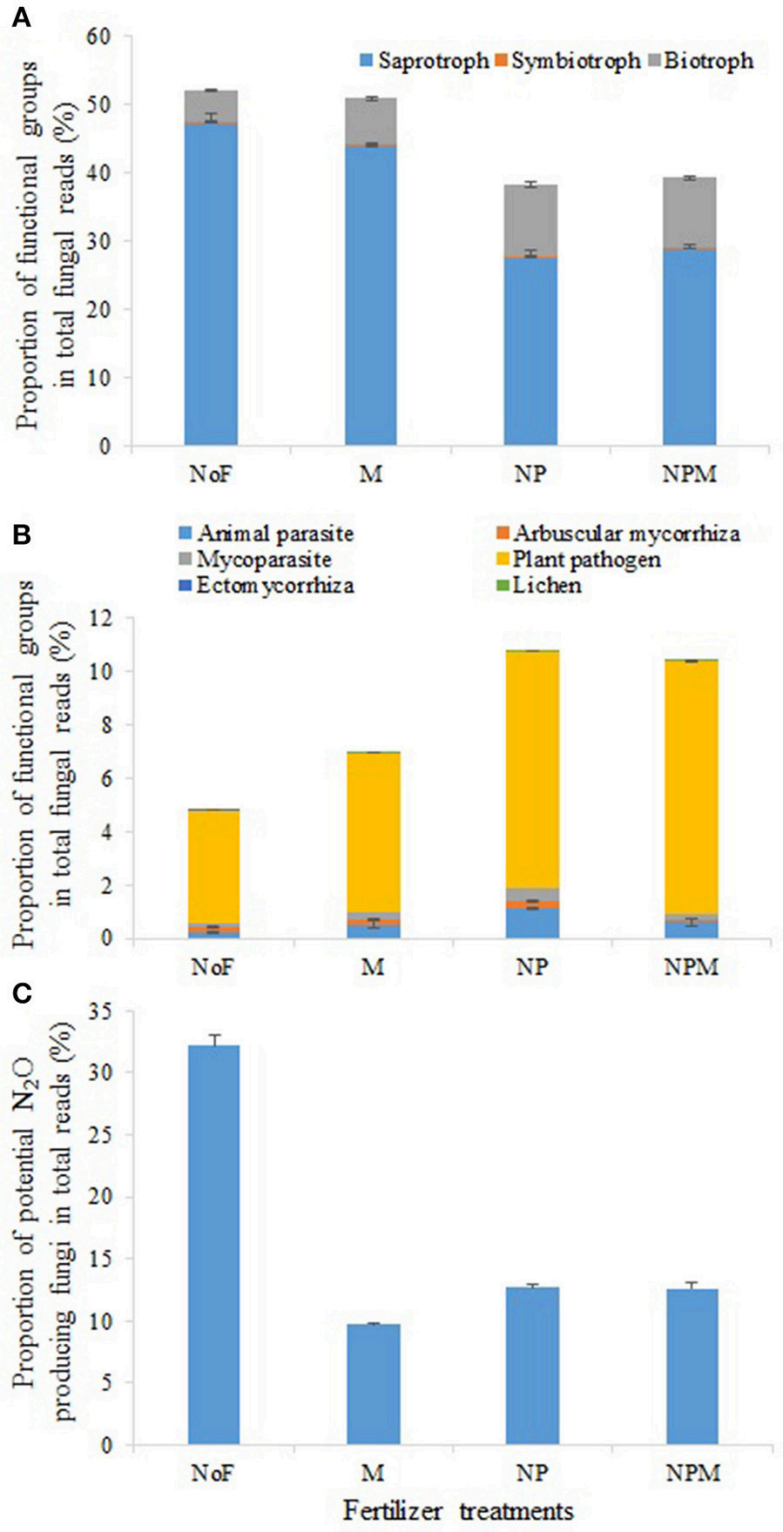
Proportional changes of major functional and ecological groups in the soil fungal community in response to 30 years of fertilizer application. Fungal functional groups with different putative trophic styles **(A)** and putative life styles **(B)** and potential N_2_O producing fungi **(C)**. Error bars are standard errors (*n* = 3). NoF, no fertilizer; M, organic manure; NP, nitrogen plus phosphorus; NPM, NP plus M.

The main fungal functional groups were present in all soils, but their proportions in the fungal community varied across treatments. Among all fungal taxa, OTUs mainly assigned to putative saprotrophs, plant pathogens, animal parasites, mycoparasites and arbuscular mycorrhizas comprised 28.1–48.7%, 0.11–1.14%, 0.06–0.53% and 0.07–0.14%, respectively (Figure 3). The putative saprotrophs contributed 47.3% of all taxa in the unfertilized soil but accounted for a relatively low proportion (27.5–28.9%) in the NP and NPM soils (Figure 3A, Supplementary Table 1). In contrast, the proportion of putative animal parasites and mycoparasites was highest in the NP soil and lowest in the unfertilized soil (Figure 3B, Supplementary Table 1). Similarly, the proportion of putative lichens was highest in the NPM soil and lowest in the unfertilized soil. Putative plant pathogens increased proportion as the following trend, NPM and NP > M > unfertilized soil.

### Indicator OTUs associated with each fertilizer soil

**Figure 4:**
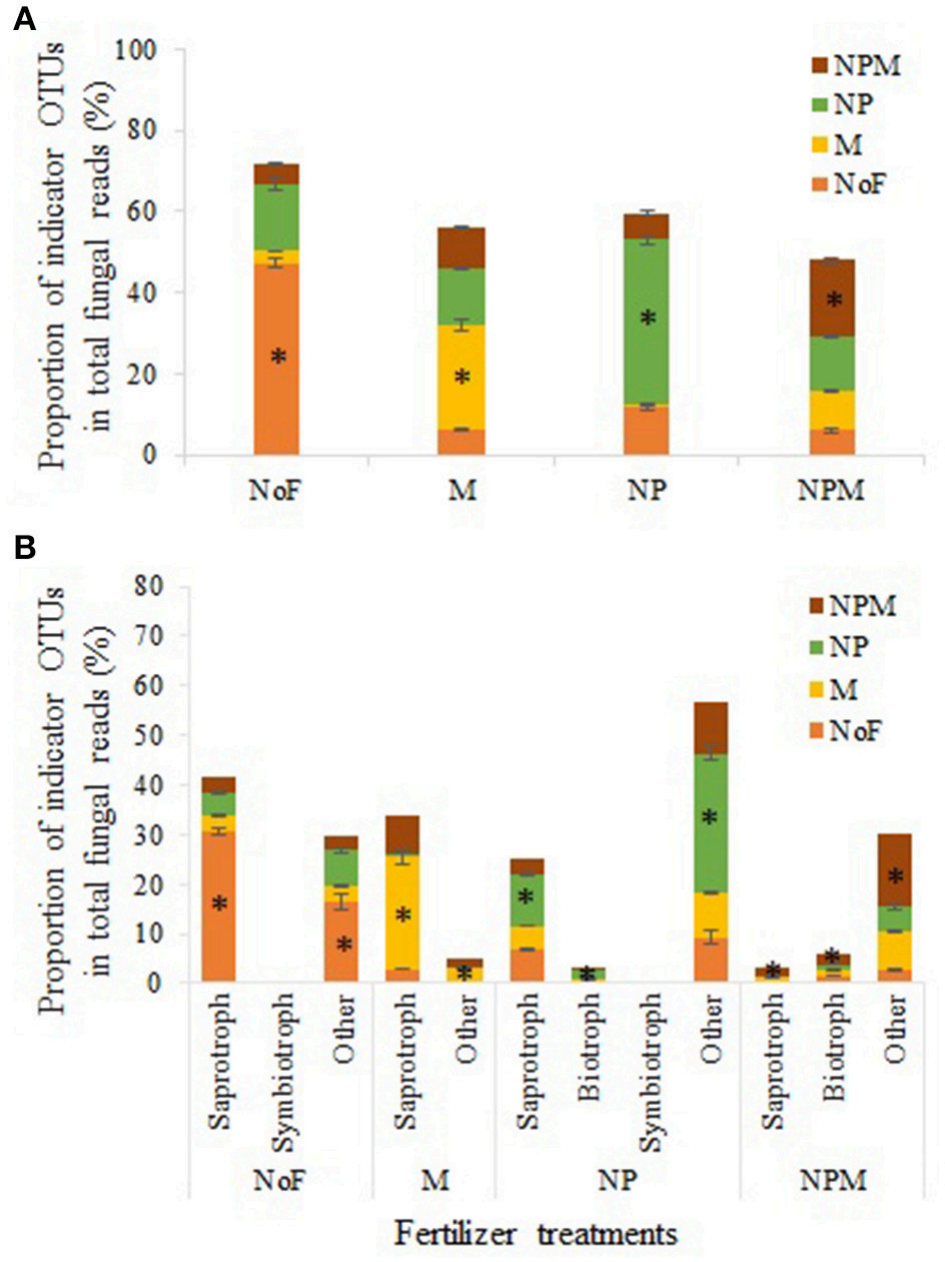
Indicator OTUs distribution in different fertilization treatments. Results of total indicator OTUs **(A)** and indicator OTUs with different putative trophic styles **(B)**. * Proportion of indicator OTUs in the treatment. Error bars are standard errors (*n* = 3). NoF, no fertilizer; M, organic manure; NP, nitrogen plus phosphorus; NPM, NP plus M.

Indicator OTUs represent ecological indicators of differences between field treatments and may reveal evidence for the impact of environmental changes induced by fertilizer treatments (De Cáceres and Legendre, 2009). Specifically, indicator species analysis (ISA) determines the strength of the association between OTUs and field treatments, and considers the relative frequency and abundance of these OTUs within each treatment (Berry et al., 2012). ISA produced an array of numerically important indicator OTUs for different field treatments (Supplementary Table 2), which are summarized in Table 4. The indicator OTUs (of the fungal community) of the NP soil occupied 14.1% in proportion in the M soil, while the indicator OTUs of the M soil accounted for only 0.9% in the NP soil (Figure 4A). Furthermore, these indicator OTUs were identified for trophic and/or life styles. The indicator OTUs classified as putative saprotrophs accounted for 30.6% in total fungal reads in the unfertilized soil, 22.6% in the M soil, 10.6% in the NP soil, and 1.8% in the NPM soil (Figure 4B). In the NP and NPM soils, the indicator OTUs were also identified as putative plant pathogen and animal parasite (Table 4). The indicator OTUs with unknown trophic categories occupied 10–28% in proportion in all soils.

**Table 4:** Treatments associated fungal indicator OTUs of highest proportion (*P* < 0.05).

**Treatment**	**Fungal genera^†^**	**Trophic style**	**Life style**
NoF	*Chaetomium, Penicillium, Myrothecium, Pyrenochaeta, Stachybotrys, Cladophialophora, Talaromyces, Podospora, Ajellomyces*	Saprotroph
	*Amanita*	Symbiotroph	Ectomycorrhiza
		
M	*Kernia, Pseudeurotium, Cladorrhinum, Coprinellus, Microascus, Phialosimplex, Aspergillus, Stropharia, Plectania*	Saprotroph
		
NP	*Trichocladium, Staphylotrichum, Cryptococcus, Gliomastix*	Saprotroph
	*Cylindrocarpon, Spizellomyces, Thanatephorus*	Biotroph	Plant pathogen
	*Metacordyceps, Beauveria*	Biotroph	Animal parasite
	*Hypocrea*	Biotroph	Mycoparasite
		
NPM	*Preussia, Acremonium, Microascus, Scopulariopsis, Myrothecium, Schizothecium*,	Saprotroph
	*Fusarium, Mycocentrospora, Gibberella*	Biotroph	Plant pathogen
	*Beauveria*	Biotroph	Animal parasite

*Fungal genera were shown for the indicator OTUs and further arranged into ecological and functional categories. (NoF, no fertilizer; M, organic manure; NP, nitrogen plus phosphorus; NPM, NP plus M)*.

† *Top 10 genera are shown here if more than 10 genera were with p < 0.05 in a treatment*.

### Correlations between soil chemical and biological parameters with fungal communities

Multiple regression model suggested that total wheat biomass, available P and respiration rate were the strongest predictors of total fungal abundance, explaining 87.6% of the variance (Table 5). Soil respiration rate was positively correlated with the proportion of putative plant pathogen but negatively correlated with putative saprotrophic fungi proportion. Consisting with this, the RDA analysis revealed that soil available P and respiration rate had the strongest influence on the community structure of total fungi and putative saprotrophs (Supplementary Figure 3). Plant pathogen community structure was significantly related to soil respiration and the DOC/AN/AP. The proportion of class Sordariomycetes was positively correlated to the concentrations of nitrate, dissolved organic C and available P, while the proportion of class Dothideomycetes was positively correlated to the C/N ratio and the respiration rate (Supplementary Table 3).

**Table 5:** Variation explained by the environmental variables in the regression models of the total fungal abundance and the proportions of fungal groups.

**Environmental variables**		**Fungal groups**					
	**Total fungi**	**Saprotrophs**	**Plant pathogen**	**Sordariomycetes**	**Dothideomycetes**	**Agaricomycetes**	**Leotiomycetes**
Wheat biomass	0.84					
Available P	0.03	0.05		0.01		0.62	0.51
${\text{NO}}_{3}^{-}$-N				0.62		
Dissolved organic C				0.01		
Microbial biomass C						0.01
Respiration	0.01	0.45	0.67		0.01	
Total N						
C/N^§^					0.43	
Adjusted *R*^2^	0.88	0.50	0.67	0.64	0.44	0.63	0.51

*Estimates of the fraction of explained variation are only reported for significant variables (P < 0.05)*.

§ *The ratios of soil total organic C to total N*.

## Discussion

### Fungal communities in unfertilized vs. fertilized soils

Long-term mineral and organic fertilizer application to the alkaline soil in this study generally increased both fungal abundance (quantified by qPCR) and fungal Simpson's diversity (Table 3). This is partly consistent with previous findings in neutral and acidic soils, where fungal abundance was enhanced but fungal diversity was reduced with N fertilization (Allison et al., 2007; Wang et al., 2015; Zhou et al., 2016). Such discrepancy is probably because the soil pH in this study was inherently different from those in previous reports. In previous studies with acid and neutral soils, N application led to soil acidification, which further changed soil fungal community (Zhou et al., 2016). However, in the alkaline soil of this study, fungal abundance and diversity showed strong correlations with soil total organic C, total N and available P concentrations (Supplementary Table 3), all of which are factors known to influence the soil microbiota (Lauber et al., 2008). Moreover, in a previous study, fungal community composition was significantly related to soil fertility (Sterkenburg et al., 2015). This indicates that fertilizer-induced increase in soil fertility might be the strong driver of fungal community in alkaline soils.

Ascomycota, who is a key decomposer in agricultural soils (Ma et al., 2013), showed no difference between mineral fertilized and unfertilized soils. This is not consistent with previous findings that Ascomycota increased proportions in N application soils (Weber et al., 2013; Zhou et al., 2016). This might be due to the different responses of fungal taxa at lower phylogenetic levels to fertilizer applications. Most members of Ascomycota were affiliated to class Sordariomycetes, which increased proportion in manure application soils compared to the unfertilized soil. The second most dominant class in Ascomycota in this study was Dothideomycetes, which in together with class Leotiomycetes increased proportions in mineral fertilized soils (Figure 2). Sordariomycetes and Leotiomycetes has been shown to increase proportion (Freedman et al., 2015; Mueller et al., 2015) but Dothideomycetes decreased with N fertilization (Freedman et al., 2015; Zhou et al., 2016). The changes of class Dothideomycetes in this study were mainly driven by the genus *Pseudogymnoascus*, which is known as saprotrophic fungi (Małecka et al., 2015). Moreover, the genus *Tetracladium* drove the changes of class Leotiomycetes in mineral fertilized soil, and is known as common root fungi (Sati et al., 2009) which has potential to benefit the growth and nutrient acquisition of their host plants. These results indicate that mineral fertilizer application in long term might enhance these taxa related litter decomposition and plant-fungal symbioses in the studied soil.

The proportion of Basidiomycota decreased in the mineral fertilizer and manure application soils and such decline in proportion was more pronounced in the M and NPM than in the NP (Figure 2). This may be related to the organic carbon composition in the M and NPM treatments. Basidiomycota was dominated by the class Agaricomycetes which are particularly important during the later stage of litter decay (Purahong et al., 2016). The high organic matter quality and input rate in the manure fertilized soils may have reduced the competitiveness of late-stage fungi. While not consistent with the increasing pattern observed in high fertility forest soils in Sterkenburg et al. (2015), the reduced Basidiomycota proportion in this study agrees well with the recent findings from soils with high N application (Weber et al., 2013; Paungfoo-Lonhienne et al., 2015; Zhou et al., 2016).

Although the proportion of several putative saprotrophic fungal genera increased in the fertilized soils, such as *Staphylotrichum* and *Trichocladium* in the M soil, *Kernia* in the NP soil and *Microascus* in the NPM soil (Supplementary Figure 2), the overall putative saprotrophs proportion was decreased in the mineral fertilized soils compared to the unfertilized soil (Figure 3). This was mainly driven by the genera *Chaetomium* and *Penicillium* whose primary niche appears to be cellulose decomposition (Li et al., 2013; Sharma et al., 2013). Since cellulose is the dominant form of carbon entering in arable soils (Jin and Chen, 2007; Thomsen et al., 2008), the decrease in putative saprotrophs proportion may contribute to the increase of soil C storage (Table 1). This is in agreement with a previous finding that mineral fertilizer reduced soil cellobiohydrolase activity in an arable soil (Fan et al., 2012). Furthermore, several saprotrophic fungal genera such as *Chaetomium* and *Myrothecium* in the class Sordariomycetes and genera *Penicillium* and *Talaromyces* in the class Eurotiomycetes, were strongly reduced by mineral and organic fertilizer applications. These genera were potential N_2_O-producing fungal denitrifiers (Mothapo et al., 2013, 2015), their overall decrease in proportion (Figure 3) indicates that fungal induced N_2_O emission might be decreased in the fertilized soils.

In contrast to putative saprotrophs, proportion of putative plant pathogens significantly increased after long-term fertilizer application (Figure 3). Its community structure was also altered, with a greater change in mineral fertilizer soil than in organic manure soil (Table 2). This is in line with several recent studies that the pathogenic taxa increased proportion after chronic fertilizer application in agricultural and forest soils (Paungfoo-Lonhienne et al., 2015; Morrison et al., 2016). Moreover, the increased proportion of putative plant pathogens did not have negative effects on plant biomass in the fertilized soil in this study (Table 1). This agrees well with other studies in agricultural soils (Morrison et al., 2016; Zhou et al., 2016), and also stays consistent with the findings in natural soils that proportion of plant pathogens was not related to plant richness (Tedersoo et al., 2014). However, it has also been suggested that plant pathogens at least partly coevolve with their hosts, as they usually attack a phylogenetically limited set of host plants (Gilbert and Webb, 2007). The specific role of plant pathogens in agricultural and natural soils still requires further investigations.

It is important to understand that we can only speculate on the ecological role of the detected taxa based on previous descriptions in other studies. Moreover, we found several management-sensitive fungal taxa which we have little or no information about their lifestyle or even their taxonomic information at lower levels. Therefore, our data should not be overgeneralized and the observations need to be confirmed in other agricultural systems.

### Management-sensitive fungal taxa in mineral fertilizer vs. organic manure application soils

The indicator OTUs in the M soil were distinctively different from those in the NP soil (Table 4, Figure 4), indicating that manure application can select or promote fungal taxa significantly different from mineral fertilizer application. Furthermore, the indicator OTUs in the M soil were mainly classified into putative saprotrophs, whilst the taxa of putative plant pathogen and animal parasite were also promoted by the NP and NPM soils. The predominance of putative saprotrophic indicator OTUs in the M soil is probably because fungal genera related to degradation processes of organic materials was closely associated with manure-based systems (Hartmann et al., 2015). The increase of putative plant pathogen in the NP and NPM soils is in line with previous findings that fungal genera with known pathogenic traits tend to increase proportions in mineral fertilizer application soils (Morrison et al., 2016; Zhou et al., 2016). Nevertheless, for most of taxa promoted by the NP and NPM soils, no information about their lifestyle or taxonomic information at lower levels is available. Hence, the ecological importance of the promoted taxa in mineral fertilizer-based soils remains to be determined. Furthermore, the varied taxa promoted by different fertilization regimes observed in this study is also in good agreement with previous findings, implying that only very few taxonomic groups responded uniformly to management practices (Hartmann et al., 2015).

### Patterns of fungal communities in the alkaline soils and possible predictors

Similar to the findings from neutral and acidic agricultural soils (Lentendu et al., 2014; Francioli et al., 2016; Zhou et al., 2016), Ascomycota and Basidiomycota were the most abundant phyla and overall proportion of putative plant pathogen increased after fertilizer application in the alkaline soils investigated in this study (Figure 2). However, the responses of fungal taxa to fertilizer application were substantially different in the alkaline soils. For instance, while Basidiomycota decreased and Ascomycota remained stable in the fertilized soils of this study, Basidiomycota remained stable and Ascomycota decreased by mineral fertilizer application in neutral and acidic agricultural soils (Francioli et al., 2016; Zhou et al., 2016). Unlike the dominant influences of soil pH in the neutral and acidic soils (Lentendu et al., 2014; Zhou et al., 2016), we found that available P and respiration rate had the strongest influence on the total abundance and community structure of soil fungi in the alkaline soil of this study (Table 5, Supplementary Figure 3). The cellulose decomposition-related saprotrophs, such as *Chaetomium* and *Penicillium*, decreased proportion in the mineral fertilizer application soils in this study, but increased in an acidic forest soil reported in Morrison et al. (2016). However, the discrepant changes of cellulose decomposition-related saprotrophs in two soils were both related to the increase of soil organic C content. This is probably because cellulose is the main form of plant input in agricultural soil (Jin and Chen, 2007; Thomsen et al., 2008) while lignin is the major source in forest soil (Frey et al., 2014). Moreover, based on observations from an agricultural soil of pH 4.6–6.4, Zhou et al. (2016) reported that mineral fertilizer application induced changes of soil fungal community had a potential negative impact on soil C storage. The discrepant observations between our study and Zhou et al. (2016) jointly highlight the necessity to further distinguish the possibly different responses of soil fungi between alkaline soil and acidic and/or neutral soil.

## Conclusion

In the alkaline soils used in this study, long-term mineral and organic fertilizer applications not only increased fungal abundance and diversity, but also altered fungal community structure. Such changes were more pronounced in the organic manure treated soils (M and NPM) than in the mineral fertilizer NP soil. In particular, mineral fertilizer application (NP and NPM) could select, promote or reduce specific groups that may have positive impacts on soil C and N cycling. Furthermore, fungal genera known as plant pathogens were better promoted by mineral fertilizer than by organic manure. The discrepant observations on fungal community between the alkaline soil in this study and previous reports from neutral and acidic soils jointly highlight the necessity to further distinguish the possibly different responses of soil fungi among different soil types.

## Author contributions

YW was responsible for the experimental design, data processing and article writing. YW and HJ collected the samples. HJ and RW contributed to the physiochemical data of soil samples. YW and YH provided essential ideas to the article writing. YH and SG contributed in reviewing the manuscript. JR contributed to the redundancy analysis.

### Conflict of interest statement

The authors declare that the research was conducted in the absence of any commercial or financial relationships that could be construed as a potential conflict of interest.
