# Supplementary material for: Different Selectivity in Fungal Communities Between Manure and Mineral Fertilizers: A Study in an Alkaline Soil After 30 Years Fertilization
Source: Front Microbiol. 2018 Oct 31;9:2613. doi: 10.3389/fmicb.2018.02613 (PMC6220076; doi:10.3389/fmicb.2018.02613)
Supplement: Supplementary file 1 [file Table_1.DOCX]

**Supplemental tables**

**Supplementary Table S1** The results of the ANOVA for fungal phyla, classes and functional groups under different fertilization regimes for 30 years in an alkaline soil.

| Variables | d.f. | SS | MS | *P* |
| --- | --- | --- | --- | --- |
| Fungal phylum |  |  |  |  |
| Ascomycota | 3 | 53.75 | 17.92 | 0.117 |
| Basidiomycota | 3 | 36.97 | 12.33 | **0.01** |
| Chytridiomycota | 3 | 1.23 | 0.41 | 0.248 |
| Zygomycota | 3 | 0.18 | 0.06 | 0.655 |
| Glomeromycota | 3 | 0.04 | 0.01 | 0.13 |
| Fungal class |  |  |  |  |
| Sordariomycetes | 3 | 240.8 | 80.30 | **0.015** |
| Dothideomycetes | 3 | 37.87 | 12.62 | **0.005** |
| Agaricomycetes | 3 | 7.63 | 2.54 | **0.011** |
| Eurotiomycetes | 3 | 83.85 | 27.95 | **0.004** |
| Leotiomycetes | 3 | 0.96 | 0.32 | **0.014** |
| Trophic style |  |  |  |  |
| Saprotroph | 3 | 930.3 | 310.1 | **<0.001** |
| Symbiotroph | 3 | 0.01 | 0.003 | 0.25 |
| Biotroph | 3 | 73.30 | 24.43 | **<0.001** |
| Life style |  |  |  |  |
| Animal parasite | 3 | 1.35 | 0.450 | **0.001** |
| Arbuscular mycorrhiza | 3 | 0.008 | 0.003 | 0.26 |
| Mycoparasite | 3 | 0.22 | 0.07 | **0.004** |
| Plant pathogen | 3 | 54.42 | 18.14 | **<0.001** |
| Ectomycorrhiza | 3 | 0.003 | 0.001 | **<0.001** |
| Lichen | 3 | 0.001 | 0.0002 | **0.002** |
| Potential N_2_O producing fungi | 3 | 960.94 | 320.31 | **<0.001** |

d.f.: degree of freedom; SS: Stdev square; MS: Mean square.

**Supplementary Table S2** OTUs associated to each treatment through indicator analysis. (NoF: no fertilizer; M: organic manure; NP: nitrogen plus phosphorus; NPM: NP plus M).

| OTU_  ID | Phylum/Class | Genus | Trophic status | Lifestyle | NoF | M | NP | NPM |
| --- | --- | --- | --- | --- | --- | --- | --- | --- |
| 1 | Sordariomycetes | Unassigned | Unknown | Unknown |  |  | • |  |
| 2 | Sordariomycetes | *Trichocladium* | Saprotroph | Unknown |  |  | • |  |
| 3 | Ascomycota | *Staphylotrichum* | Saprotroph | Unknown |  |  | • |  |
| 4 | Sordariomycetes | *Chaetomium* | Saprotroph | Unknown | • |  |  |  |
| 5 | Sordariomycetes | Unassigned | Unknown | Unknown |  |  |  | • |
| 6 | Sordariomycetes | Unassigned | Unknown | Unknown |  |  | • |  |
| 8 | Sordariomycetes | *Kernia* | Saprotroph | Unknown |  | • |  |  |
| 12 | Sordariomycetes | Unassigned | Unknown | Unknown |  |  |  | • |
| 14 | Dothideomycetes | Unassigned | Unknown | Unknown |  |  | • |  |
| 15 | Sordariomycetes | *Fusarium* | Biotroph | Plant pathogen |  |  |  | • |
| 22 | Tremellomycetes | *Cryptococcus* | Saprotroph | Unknown |  |  | • |  |
| 23 | Dothideomycetes | Unassigned | Unknown | Unknown | • |  |  |  |
| 24 | Eurotiomycetes | *Penicillium* | Saprotroph | Unknown | • |  |  |  |
| 25 | Unassigned | Unassigned | Unknown | Unknown |  |  | • |  |
| 28 | Unassigned | Unassigned | Unknown | Unknown |  |  |  | • |
| 30 | Sordariomycetes | Unassigned | Unknown | Unknown |  | • |  |  |
| 34 | Dothideomycetes | *Preussia* | Saprotroph | Unknown |  |  |  | • |
| 36 | Eurotiomycetes | Unassigned | Unknown | Unknown | • |  |  |  |
| 37 | Ascomycota | Unassigned | Unknown | Unknown |  |  |  | • |
| 41 | Sordariomycetes | Unassigned | Unknown | Unknown |  |  |  | • |
| 47 | Sordariomycetes | Unassigned | Unknown | Unknown |  |  | • |  |
| 49 | Dothideomycetes | Unassigned | Unknown | Unknown | • |  |  |  |
| 62 | Sordariomycetes | *Cylindrocarpon* | Biotroph | Plant pathogen |  |  | • |  |
| 63 | Sordariomycetes | Unassigned | Unknown | Unknown |  |  | • |  |
| 65 | Sordariomycetes | *Fusarium* | Biotroph | Plant pathogen |  |  |  | • |
| 68 | Dothideomycetes | *Mycocentrospora* | Biotroph | Plant pathogen |  |  |  | • |
| 71 | Eurotiomycetes | Unassigned | Unknown | Unknown |  |  | • |  |
| 72 | Eurotiomycetes | Unassigned | Unknown | Unknown | • |  |  |  |
| 74 | Unassigned | Unassigned | Unknown | Unknown |  |  | • |  |
| 76 | Sordariomycetes | *Myrothecium* | Saprotroph | Unknown | • |  |  |  |
| 78 | Agaricomycetes | Unassigned | Unknown | Unknown |  |  |  | • |
| 79 | Sordariomycetes | *Acremonium* | Saprotroph | Unknown |  |  |  | • |
| 80 | Eurotiomycetes | *Arachnomyces* | Unknown | Unknown |  |  |  | • |
| 81 | Chytridiomycetes | *Spizellomyces* | Biotroph | Plant pathogen |  |  | • |  |
| 89 | Sordariomycetes | *Metacordyceps* | Biotroph | Animal parasite |  |  | • |  |
| 90 | Agaricomycetes | Unassigned | Unknown | Unknown | • |  |  |  |
| 91 | Ascomycota | Unassigned | Unknown | Unknown | • |  |  |  |
| 92 | Dothideomycetes | *Pyrenochaeta* | Saprotroph | Unknown | • |  |  |  |
| 97 | Ascomycota | Unassigned | Unknown | Unknown | • |  |  |  |
| 99 | Sordariomycetes | Unassigned | Unknown | Unknown | • |  |  |  |
| 101 | Agaricomycetes | Unassigned | Unknown | Unknown | • |  |  |  |
| 102 | Sordariomycetes | *Stachybotrys* | Saprotroph | Unknown | • |  |  |  |
| 103 | Sordariomycetes | Unassigned | Unknown | Unknown |  |  |  | • |
| 104 | Dothideomycetes | *Pseudeurotium* | Saprotroph | Unknown |  | • |  |  |
| 106 | Eurotiomycetes | *Cladophialophora* | Saprotroph | Unknown | • |  |  |  |
| 107 | Unassigned | Unassigned | Unknown | Unknown | • |  |  |  |
| 108 | Dothideomycetes | Unassigned | Unknown | Unknown |  | • |  |  |
| 112 | Sordariomycetes | *Cladorrhinum* | Saprotroph | Unknown |  | • |  |  |
| 113 | Sordariomycetes | *Microascus* | Saprotroph | Unknown |  |  |  | • |
| 115 | Eurotiomycetes | *Talaromyces* | Saprotroph | Unknown | • |  |  |  |
| 117 | Sordariomycetes | Unassigned | Unknown | Unknown |  |  |  | • |
| 119 | Sordariomycetes | Unassigned | Unknown | Unknown |  |  |  | • |
| 121 | Agaricomycetes | Unassigned | Unknown | Unknown | • |  |  |  |
| 126 | Agaricomycetes | Unassigned | Unknown | Unknown | • |  |  |  |
| 127 | Sordariomycetes | Unassigned | Unknown | Unknown | • |  |  |  |
| 129 | Agaricomycetes | *Coprinellus* | Saprotroph | Unknown |  | • |  |  |
| 131 | Sordariomycetes | *Gibberella* | Biotroph | Plant pathogen |  |  |  | • |
| 136 | Orbiliomycetes | Unassigned | Unknown | Unknown |  |  |  | • |
| 138 | Sordariomycetes | *Microascus* | Saprotroph | Unknown |  | • |  |  |
| 142 | Unassigned | Unassigned | Unknown | Unknown | • |  |  |  |
| 144 | Sordariomycetes | *Colletotrichum* | Biotroph | Plant pathogen |  |  |  | • |
| 148 | Monoblepharidomycetes | *Harpochytrium* | Unknown | Unknown | • |  |  |  |
| 149 | Dothideomycetes | *Phaeosphaeria* | Saprotroph | Unknown |  |  | • |  |
| 150 | Agaricomycetes | *Myriococcum* | Unknown | Unknown |  | • |  |  |
| 153 | Unassigned | Unassigned | Unknown | Unknown | • |  |  |  |
| 157 | Sordariomycetes | *Scopulariopsis* | Saprotroph | Unknown |  |  |  | • |
| 159 | Agaricomycetes | Unassigned | Unknown | Unknown | • |  |  |  |
| 160 | Sordariomycetes | *Hypocrea* | Biotroph | Mycoparasite |  |  | • |  |
| 163 | Sordariomycetes | *Myrothecium* | Saprotroph | Unknown |  |  |  | • |
| 167 | Dothideomycetes | Unassigned | Unknown | Unknown | • |  |  |  |
| 168 | Agaricomycetes | Unassigned | Unknown | Unknown | • |  |  |  |
| 170 | Agaricomycetes | Unassigned | Unknown | Unknown | • |  |  |  |
| 176 | Unassigned | Unassigned | Unknown | Unknown |  |  | • |  |
| 180 | Ascomycota | Unassigned | Unknown | Unknown |  |  | • |  |
| 181 | Agaricomycetes | *Thanatephorus* | Biotroph | Plant pathogen |  |  | • |  |
| 183 | Agaricomycetes | Unassigned | Unknown | Unknown | • |  |  |  |
| 189 | Sordariomycetes | *Zopfiella* | Saprotroph | Unknown |  |  |  | • |
| 193 | Sordariomycetes | Unassigned | Unknown | Unknown |  |  |  | • |
| 205 | Sordariomycetes | *Schizothecium* | Saprotroph | Unknown |  |  |  | • |
| 208 | Ascomycota | Unassigned | Unknown | Unknown |  |  |  | • |
| 220 | Pezizomycetes | Unassigned | Unknown | Unknown |  | • |  |  |
| 221 | Dothideomycetes | *Preussia* | Saprotroph | Unknown |  |  |  | • |
| 229 | Sordariomycetes | Unassigned | Unknown | Unknown |  |  |  | • |
| 231 | Eurotiomycetes | *Arachnomyces* | Unknown | Unknown |  |  |  | • |
| 232 | Sordariomycetes | Unassigned | Unknown | Unknown |  | • |  |  |
| 238 | Sordariomycetes | Unassigned | Unknown | Unknown |  | • |  |  |
| 245 | Sordariomycetes | Unassigned | Unknown | Unknown |  |  |  | • |
| 249 | Unassigned | Unassigned | Unknown | Unknown | • |  |  |  |
| 251 | Agaricomycetes | Unassigned | Unknown | Unknown | • |  |  |  |
| 252 | Sordariomycetes | *Sordaria* | Saprotroph | Unknown |  | • |  |  |
| 253 | Eurotiomycetes | *Phialosimplex* | Saprotroph | Unknown |  | • |  |  |
| 260 | Sordariomycetes | *Podospora* | Saprotroph | Unknown | • |  |  |  |
| 262 | Eurotiomycetes | *Aspergillus* | Saprotroph | Unknown |  | • |  |  |
| 263 | Ascomycota | Unassigned | Unknown | Unknown |  |  |  | • |
| 266 | Glomeromycetes | *Archaeospora* | Symbiotroph | Arbuscular mycorrhiza |  |  | • |  |
| 276 | Unassigned | Unassigned | Unknown | Unknown | • |  |  |  |
| 278 | Sordariomycetes | *Beauveria* | Biotroph | Animal parasite |  |  |  | • |
| 281 | Eurotiomycetes | Unassigned | Unknown | Unknown | • |  |  |  |
| 283 | Sordariomycetes | *Myrothecium* | Saprotroph | Unknown | • |  |  |  |
| 286 | Sordariomycetes | Unassigned | Unknown | Unknown |  |  |  | • |
| 290 | Agaricomycetes | Unassigned | Unknown | Unknown | • |  |  |  |
| 291 | Sordariomycetes | *Beauveria* | Biotroph | Animal parasite |  |  | • |  |
| 293 | Agaricomycetes | *Stropharia* | Saprotroph | Unknown |  | • |  |  |
| 309 | Sordariomycetes | Unassigned | Unknown | Unknown |  |  |  | • |
| 310 | Unassigned | Unassigned | Unknown | Unknown |  |  |  | • |
| 323 | Sordariomycetes | *Microascus* | Saprotroph | Unknown |  |  |  | • |
| 327 | Dothideomycetes | Unassigned | Unknown | Unknown |  |  | • |  |
| 334 | Ascomycota | *Ciliophora* | Unknown | Unknown |  |  | • |  |
| 335 | Eurotiomycetes | Unassigned | Unknown | Unknown |  | • |  |  |
| 336 | Ascomycota | *Scolecobasidium* | Saprotroph | Unknown |  |  | • |  |
| 340 | Sordariomycetes | *Acremonium* | Saprotroph | Unknown |  |  | • |  |
| 342 | Eurotiomycetes | *Chrysosporium* | Saprotroph | Unknown |  |  |  | • |
| 347 | Ascomycota | Unassigned | Unknown | Unknown |  |  |  | • |
| 350 | Eurotiomycetes | *Ajellomyces* | Saprotroph | Unknown | • |  |  |  |
| 364 | Agaricomycetes | Unassigned | Unknown | Unknown | • |  |  |  |
| 365 | Agaricomycetes | Unassigned | Unknown | Unknown | • |  |  |  |
| 368 | Dothideomycetes | *Preussia* | Saprotroph | Unknown |  |  |  | • |
| 371 | Pezizomycetes | *Plectania* | Saprotroph | Unknown |  | • |  |  |
| 382 | Dothideomycetes | Unassigned | Unknown | Unknown | • |  |  |  |
| 389 | Sordariomycetes | *Gliomastix* | Saprotroph | Unknown |  |  | • |  |
| 392 | Orbiliomycetes | Unassigned | Unknown | Unknown |  | • |  |  |
| 402 | Sordariomycetes | *Stephanonectria* | Saprotroph | Unknown |  |  | • |  |
| 422 | Unassigned | Unassigned | Unknown | Unknown |  |  |  | • |
| 431 | Sordariomycetes | Unassigned | Unknown | Unknown |  |  |  | • |
| 440 | Agaricomycetes | Unassigned | Unknown | Unknown | • |  |  |  |
| 443 | Unassigned | Unassigned | Unknown | Unknown |  |  | • |  |
| 447 | Eurotiomycetes | *Emmonsia* | Saprotroph | Unknown | • |  |  |  |
| 456 | Dothideomycetes | Unassigned | Unknown | Unknown |  |  | • |  |
| 457 | Eurotiomycetes | *Arthroderma* | Saprotroph | Unknown |  |  |  | • |
| 491 | Sordariomycetes | *Chaetomium* | Saprotroph | Unknown | • |  |  |  |
| 499 | Agaricomycetes | *Leucoagaricus* | Saprotroph | Unknown |  | • |  |  |
| 508 | Sordariomycetes | *Volutella* | Biotroph | Plant pathogen |  |  |  | • |
| 512 | Ascomycota | Unassigned | Unknown | Unknown |  |  |  | • |
| 515 | Unassigned | Unassigned | Unknown | Unknown | • |  |  |  |
| 517 | Agaricomycetes | Unassigned | Unknown | Unknown |  |  | • |  |
| 519 | Unassigned | Unassigned | Unknown | Unknown | • |  |  |  |
| 522 | Dothideomycetes | *Gymnostellatospora* | Saprotroph | Unknown |  | • |  |  |
| 540 | Sordariomycetes | *Acremonium* | Saprotroph | Unknown |  | • |  |  |
| 547 | Sordariomycetes | Unassigned | Unknown | Unknown | • |  |  |  |
| 559 | Dothideomycetes | *Stagonospora* | Biotroph | Plant pathogen |  |  |  | • |
| 597 | Dothideomycetes | Unassigned | Unknown | Unknown |  |  |  | • |
| 618 | Agaricomycetes | *Amanita* | Symbiotroph | Ectomycorrhiza | • |  |  |  |
| 622 | Dothideomycetes | Unassigned | Unknown | Unknown | • |  |  |  |
| 626 | Unassigned | Unassigned | Unknown | Unknown | • |  |  |  |
| 632 | Sordariomycetes | *Chaetomium* | Saprotroph | Unknown | • |  |  |  |
| 722 | Sordariomycetes | *Acremonium* | Saprotroph | Unknown |  | • |  |  |
| 737 | Chytridiomycetes | Unassigned | Unknown | Unknown |  | • |  |  |
| 749 | Dothideomycetes | *Lophiostoma* | Saprotroph | Unknown |  | • |  |  |
| 763 | Agaricomycetes | Unassigned | Unknown | Unknown |  |  |  | • |
| 769 | Dothideomycetes | *Preussia* | Saprotroph | Unknown |  | • |  |  |
| 820 | Unassigned | Unassigned | Unknown | Unknown | • |  |  |  |
| 851 | Agaricomycetes | Unassigned | Unknown | Unknown | • |  |  |  |
| 887 | Sordariomycetes | Unassigned | Unknown | Unknown |  |  |  | • |
| 980 | Leotiomycetes | *Unguicularia* | Saprotroph | Unknown |  | • |  |  |
| 981 | Sordariomycetes | Unassigned | Unknown | Unknown |  |  |  | • |
| 1186 | Eurotiomycetes | *Chrysosporium* | Saprotroph | Unknown |  |  |  | • |
| 1234 | Sordariomycetes | *Podospora* | Saprotroph | Unknown | • |  |  |  |
| 1283 | Unassigned | Unassigned | Unknown | Unknown | • |  |  |  |
| 1344 | Agaricomycetes | Unassigned | Unknown | Unknown | • |  |  |  |
| 1513 | Dothideomycetes | Unassigned | Unknown | Unknown |  | • |  |  |
| 1746 | Agaricomycetes | Unassigned | Unknown | Unknown | • |  |  |  |
| 1960 | Sordariomycetes | Unassigned | Unknown | Unknown | • |  |  |  |
| 2061 | Sordariomycetes | *Podospora* | Saprotroph | Unknown | • |  |  |  |
| Total number |  |  |  |  | 57 | 27 | 30 | 49 |

**Supplementary Table S3** Spearman correlations of soil chemical and microbial variables with fungal abundance, ratio of bacteria to fungi, fungal diversity index and proportion of fungal groups.

|  | Spearman correlation coefficient (r) ^‡^ | | | | | | | | | | | |
| --- | --- | --- | --- | --- | --- | --- | --- | --- | --- | --- | --- | --- |
|  | Total C^†^ | Total N^†^ | C/N^†^ | NO_3_^-^-N | DOC | MBC^†^ | MBN^†^ | RP^†^ | AP^†^ | DOC/AN^†^ | DOC/AN/AP | Wheat biomass |
| ITS | **0.825**** | **0.804**** | 0.315 | **0.664*** | **0.818**** | **0.734**** | **0.769**** | **0.748**** | **0.741**** | 0.385 | **-0.706*** | **0.930**** |
| Bac/fungi | 0.070 | 0.112 | 0.510 | -0.210 | -0.126 | -0.070 | -0.182 | 0.119 | -0.140 | -0.224 | 0.175 | 0.014 |
| Simpson diversity index | **0.685*** | **0.657*** | -.070 | **-0.615*** | .091 | .175 | .357 | **0.755**** | 0.538 | 0.448 | -0.476 | **0.580*** |
| Shannon diversity index | **0.643^*^** | **0.692^*^** | 0.427 | 0.462 | **0.657^*^** | **0.664^*^** | **0.636^*^** | **0.720^**^** | **0.678^*^** | 0.210 | **-0.594^*^** | **0.720^**^** |
| Saprotroph | **-0.622*** | **-0.671*** | **-0.608*** | -0.154 | -0.371 | -0.371 | -0.413 | **-0.853**** | **-0.590*** | **-0.594*** | 0.343 | **-0.587*** |
| Plant pathogen | **0.713**** | **0.748**** | **0.650*** | 0.350 | 0.531 | 0.559 | 0.503 | **0.867**** | **0.587*** | 0.427 | -0.524 | **0.692*** |
| Sordariomycetes | 0.469 | 0.406 | -0.168 | **0.790**** | **0.699*** | **0.678*** | **0.664*** | 0.259 | **0.734**** | -0.049 | **-0.769**** | 0.538 |
| Dothideomycetes | 0.441 | 0.497 | **0.839**** | -0.175 | 0.014 | 0.091 | 0.077 | **0.587*** | -0.021 | 0.231 | 0.063 | 0.266 |
| Eurotiomycetes | -0.217 | -0.217 | 0.091 | -0.315 | -0.273 | -0.510 | -0.510 | -0.294 | -0.559 | -0.455 | 0.538 | -0.273 |
| Agaricomycetes | **-0.846**** | **-0.832**** | -0.503 | **-0.664*** | **-0.741**** | **-0.776**** | **-0.734**** | **-0.769**** | **-0.783**** | -0.175 | **0.769**** | **-0.776**** |
| Leotiomycetes | 0.497 | 0.517 | 0.399 | 0.266 | 0.378 | 0.545 | 0.420 | **0.678*** | **0.580*** | 0.392 | -0.552 | 0.566 |

^†^ TOC: total organic C; TN: total N; C/N: ratio of total organic C to total N; DOC: dissolved organic C; MBC: microbial biomass C; MBN: microbial biomass N; RP: respiration rate; AP: available P; AN, the sum of ammonium and nitrate.

^‡^Significance: ** *P* < 0.01; * *P* < 0.05.

**Supplemental figures**

**Supplementary Figure S1** PCA analysis of soil chemical and microbial variables under different treatments in 30-year fertilization trials. Total C: total organic C; C/N: ratios of total organic C to total N; DOC: dissolved organic C; MBC: microbial biomass C; MBN: microbial biomass N; RP: soil respiration; *Q*co_2_: respiration quotient; AP: available inorganic P; AN/AP: ratios of available inorganic N to available inorganic P; DOC/AN/AP: ratios of DOC:AN:AP. NoF: no fertilizer; M: organic manure; NP: nitrogen plus phosphorus; NPM: NP plus M.

**Supplementary Figure S2** Proportional changes of dominant fungal genera in the soil fungal community in response to 30 years of fertilizer application in an alkaline soil. Error bars are standard errors (*n* = 3). Different letters indicate significant difference at *P* < 0.05 among no fertilizer and fertilizer application soils. NoF: no fertilizer; M: organic manure; NP: nitrogen plus phosphorus; NPM: NP plus M.

**Supplementary Figure S3** Redundancy analysis of soil fungi and dominant fungal functional groups. (A) Total fungi; (B) Putative saprotrophic fungi; (C) Putative plant pathogen. MBC: microbial biomass C; MBN: microbial biomass N; Total C: total organic C; RP: soil respiration; Qco_2_: respiration quotient; C/N: the ratios of total organic C to total N; AP: available inorganic P; AN/AP: ratios of available inorganic N to available inorganic P; DOC: dissolved organic C; DOC/AN/AP: the ratios of DOC:AN:AP. NoF: no fertilizer; M: organic manure; NP: nitrogen plus phosphorus; NPM: NP plus M.
